# Infodemics and health misinformation: a systematic review of reviews

**DOI:** 10.2471/BLT.21.287654

**Published:** 2022-06-30

**Authors:** Israel Júnior Borges do Nascimento, Ana Beatriz Pizarro, Jussara M Almeida, Natasha Azzopardi-Muscat, Marcos André Gonçalves, Maria Björklund, David Novillo-Ortiz

**Affiliations:** a School of Medicine and University Hospital, Federal University of Minas Gerais, Belo Horizonte, Brazil.; b Clinical Research Center, Fundación Valle del Lili, Cali, Colombia.; c Department of Computer Science, Institute of Exact Science, Federal University of Minas Gerais, Brazil.; d Division of Country Health Policies and Systems, World Health Organization Regional Office for Europe, UN City, Marmorvej 51, 2100 Copenhagen, Denmark.; e Faculty of Medicine, Lund University, Lund, Sweden.

## Abstract

**Objective:**

To compare and summarize the literature regarding infodemics and health misinformation, and to identify challenges and opportunities for addressing the issues of infodemics.

**Methods:**

We searched MEDLINE®, Embase®, Cochrane Library of Systematic Reviews, Scopus and Epistemonikos on 6 May 2022 for systematic reviews analysing infodemics, misinformation, disinformation and fake news related to health. We grouped studies based on similarity and retrieved evidence on challenges and opportunities. We used the AMSTAR 2 approach to assess the reviews’ methodological quality. To evaluate the quality of the evidence, we used the Grading of Recommendations Assessment, Development and Evaluation guidelines.

**Findings:**

Our search identified 31 systematic reviews, of which 17 were published. The proportion of health-related misinformation on social media ranged from 0.2% to 28.8%. Twitter, Facebook, YouTube and Instagram are critical in disseminating the rapid and far-reaching information. The most negative consequences of health misinformation are the increase of misleading or incorrect interpretations of available evidence, impact on mental health, misallocation of health resources and an increase in vaccination hesitancy. The increase of unreliable health information delays care provision and increases the occurrence of hateful and divisive rhetoric. Social media could also be a useful tool to combat misinformation during crises. Included reviews highlight the poor quality of published studies during health crises.

**Conclusion:**

Available evidence suggests that infodemics during health emergencies have an adverse effect on society. Multisectoral actions to counteract infodemics and health misinformation are needed, including developing legal policies, creating and promoting awareness campaigns, improving health-related content in mass media and increasing people’s digital and health literacy.

## Résumé

**Objectif:**

Comparer et synthétiser la littérature consacrée à l'infodémie et à la désinformation sanitaire, mais aussi identifier les défis et opportunités inhérents à la lutte contre cette problématique.

**Méthodes:**

Nous avons exploré les bases de données MEDLINE®, Embase®, Cochrane Library of Systematic Reviews, Scopus et Epistemonikos le 6 mai 2022 à la recherche de revues systématiques analysant les infodémies, la désinformation, les fausses informations et les «fake news» liées à la santé. Nous avons ensuite regroupé les études en fonction de leurs similitudes et en avons extrait des éléments probants relatifs aux défis et opportunités. Nous avons employé l'approche AMSTAR-2 afin de mesurer la qualité méthodologique des différentes revues. Enfin, pour évaluer la qualité des éléments probants, nous avons utilisé les critères du système GRADE (Grading of Recommendations, Assessment, Development and Evaluation, soit «grade donné aux recommandations, examen, élaboration et évaluation»).

**Résultats:**

Nos recherches nous ont permis de dénicher 31 revues systématiques, dont 17 ont été publiées. Sur les réseaux sociaux, le pourcentage d'informations fallacieuses concernant la santé était compris entre 0,2 et 28,8%. Twitter, Facebook, YouTube et Instagram jouent un rôle prépondérant dans la propagation rapide d'informations à grande échelle. Cette désinformation entraîne de multiples conséquences négatives: hausse du nombre d'interprétations erronées ou trompeuses des preuves existantes, impact sur la santé mentale, mauvaise affectation des ressources en santé et méfiance croissante vis-à-vis de la vaccination. La prolifération des informations sanitaires non fiables retarde la prise en charge tout en alimentant les réticences et les discours clivants. Néanmoins, les réseaux sociaux peuvent aussi se révéler utiles dans la lutte contre la désinformation lors des crises. Les revues examinées soulignent la qualité médiocre des études publiées durant les crises sanitaires.

**Conclusion:**

Tout porte à croire que les infodémies qui surgissent dans le cadre des urgences sanitaires sont néfastes pour la société. Des actions multisectorielles sont nécessaires pour combattre les fausses informations, notamment le développement de politiques juridiques, l'élaboration et le déploiement de campagnes de sensibilisation, l'amélioration des contenus dédiés à la santé dans les médias de masse, et une meilleure éducation à la culture numérique et à la santé.

## Resumen

**Objetivo:**

Comparar y resumir la literatura relacionada con la infodemia y la información errónea sobre la salud, e identificar los desafíos y las oportunidades para abordar los problemas de la infodemia.

**Métodos:**

Se realizaron búsquedas en MEDLINE®, Embase®, la Biblioteca Cochrane de Revisiones Sistemáticas, Scopus y Epistemonikos el 6 de mayo de 2022 para obtener revisiones sistemáticas que analizaran la infodemia, la información errónea, la desinformación y las noticias falsas relacionadas con la salud. Se agruparon los estudios en función de la similitud y se recuperaron las pruebas sobre los desafíos y las oportunidades. Se utilizó el enfoque AMSTAR-2 para valorar la calidad metodológica de las revisiones. Además, para evaluar la calidad de las pruebas, se utilizaron los criterios del sistema GRADE (Grading of Recommendations Assessment, Development and Evaluation, o bien, el grado asignado a las recomendaciones, la valoración, el desarrollo y la evaluación).

**Resultados:**

Nuestra búsqueda identificó 31 revisiones sistemáticas, de las que 17 estaban publicadas. El porcentaje de información errónea relacionada con la salud en las redes sociales osciló entre el 0,2 y el 28,8 %. Twitter, Facebook, YouTube e Instagram son fundamentales en la difusión de la información rápida y de gran alcance. Las consecuencias más negativas de la información errónea sobre la salud son el aumento de las interpretaciones engañosas o incorrectas de las pruebas disponibles, el impacto en la salud mental, la asignación inadecuada de los recursos sanitarios y el aumento de las dudas sobre la vacunación. El aumento de la información sanitaria poco fiable retrasa la prestación de cuidados y aumenta la aparición de una retórica de rechazo y división. Por otra parte, los medios sociales podrían ser una herramienta útil para combatir la información errónea durante las crisis. Las revisiones incluidas destacan la mala calidad de los estudios publicados durante las crisis sanitarias.

**Conclusión:**

Las pruebas disponibles sugieren que la infodemia durante las emergencias sanitarias tiene un efecto adverso en la sociedad. Se necesitan acciones multisectoriales para contrarrestar la infodemia y la información errónea sobre la salud, como el desarrollo de políticas legales, la creación y promoción de campañas de sensibilización, la mejora de los contenidos relacionados con la salud en los medios de comunicación y el aumento de la alfabetización digital y sanitaria de la población.

## ملخص

**الغرض:**

مقارنة وتلخيص المنشورات المتعلقة بالمعلومات غير الدقيقة والمعلومات الصحية الخاطئة، وتحديد التحديات والفرص لمواجهة مشكلات المعلومات غير الدقيقة.

**الطريقة:**

قمنا بالبحث في MEDLINE®‎، وEmbase®‎، ومكتبة Cochrane للمراجعات المنهجية، وScopus، وEpistemonikos، في 6 مايو/أيار 2022 عن المراجعات المنهجية التي تحلل المعلومات غير الدقيقة، والمعلومات الخاطئة، والمعلومات المضللة، والأخبار الملفقة المتعلقة بالصحة. قمنا بتجميع الدراسات على أساس التشابه، واسترجعنا الأدلة على التحديات والفرص. وقمنا بالاستعانة بأسلوب AMSTAR-2 لتقييم الجودة المنهجية للمراجعات. لتقييم جودة الأدلة، قمنا بالاستعانة بتصنيف تقييم التوصيات (Grading of Recommendations Assessment)، وبالإرشادات الخاصة بالتطوير والتقييم (Development and Evaluation).

**النتائج:**

حدد البحث الذي قمنا به عدد 31 مراجعة منهجية، تم نشر 17 منها. تراوحت نسبة المعلومات الخاطئة المتعلقة بالصحة على وسائل التواصل الاجتماعي من 0.2% إلى 28.8%. تعد كل من منصات Twitter، وFacebook، وYouTube، وInstagram، منصات أساسية في نشر المعلومات السريعة وبعيدة الانتشار. تتمثل أكثر العواقب السلبية للمعلومات الصحية الخاطئة في زيادة التفسيرات المضللة أو غير الصحيحة للأدلة المتاحة، والتأثير على الصحة العقلية، وسوء تخصيص الموارد الصحية، وزيادة في التردد بخصوص التحصين. تؤدي زيادة المعلومات الصحية غير الموثوقة إلى تأخير تقديم الرعاية، وتزيد من حدوث خطاب الكراهية والانقسام. يمكن أن تكون وسائل التواصل الاجتماعي أيضًا أداة مفيدة لمواجهة المعلومات الخاطئة أثناء الأزمات. تسلط المراجعات المتضمنة الضوء على الجودة المنخفضة للدراسات المنشورة أثناء الأزمات الصحية.

**الاستنتاج:**

تشير الأدلة المتاحة إلى أن المعلومات غير الدقيقة أثناء حالات الطوارئ الصحية لها تأثير سلبي على المجتمع. هناك حاجة إلى إجراءات متعددة القطاعات لمواجهة المعلومات غير الدقيقة والمعلومات الصحية الخاطئة، بما في ذلك تطوير السياسات القانونية، وإطلاق حملات للتوعية وترويجها، وتحسين المحتوى المتعلق بالصحة في وسائل الإعلام، وزيادة المعرفة الرقمية والصحية للأشخاص.

## 摘要

**目的:**

比较和总结与信息流行病和健康错误信息有关的文献,并确定在解决信息流行病问题方面所面临的挑战和机遇。

**方法:**

我们已于 2022 年 5 月 6 日搜索了 MEDLINE®、Embase®、Cochrane 系统评价图书馆、Scopus 和 Epistemonikos,通过分析信息流行病以及与健康相关的错误信息、虚假信息和假新闻,完成了系统评价。我们基于相似性对研究进行了分组,并检索了与挑战和机遇有关的证据。我们使用 AMSTAR-2 方法来评估审查的方法学质量。为了评估证据的质量,我们使用了《推荐意见评估、制定和评价分级指南》。

**结果:**

经搜索,我们发现了 31 篇系统评价,其中 17 篇已发表。社交媒体上健康相关错误信息的比例占 0.2% 至 28.8% 不等。推特网 (Twitter)、脸书 (Facebook)、YouTube 和 Instagram 是致使信息得以快速传播并造成深远影响的重要渠道。健康错误信息导致的最严重负面影响是对现有证据的误导或错误理解进一步加剧、对心理健康造成不利影响、导致卫生资源分配错误以及导致疫苗接种犹豫人群的比例增加。不可靠健康信息的增加导致护理服务延迟提供且反对和分裂言论增多。社交媒体也可成为在危机期间打击错误信息的有用工具。有些评论强调,健康危机期间所公布的研究质量较差。

**结论:**

现有证据表明,卫生突发事件期间信息流行病对社会产生了不利影响。需要多个部门共同行动以抵制信息流行病和健康错误信息,包括制定法律政策、创建和推广意识活动、加强对大众媒体健康相关内容的管理以及提高人们的数字和健康素养。

## Резюме

**Цель:**

Сопоставить и обобщить литературу по инфодемии и дезинформации в области здравоохранения, а также определить сложные задачи и возможности для решения проблем инфодемии.

**Методы:**

6 мая 2022 г. авторы выполнили поиск информации в базе данных MEDLINE®, Embase®, Cochrane Library of Systematic Reviews, Scopus и Epistemonikos на предмет систематических обзоров, анализирующих инфодемию, ложную информацию, дезинформацию и фейковые новости о здравоохранении. Авторы сгруппировали исследования на основе сходства и получили данные о сложных задачах и возможностях. Авторы использовали подход AMSTAR-2 для оценки методологического качества обзоров. Для оценки качества данных авторы использовали Руководство по ранжированию оценки, разработки и экспертизы рекомендаций.

**Результаты:**

Поиск выявил 31 систематический обзор, 17 из которых были опубликованы. Доля дезинформации в области здравоохранения в социальных сетях колебалась от 0,2 до 28,8%. Twitter, Facebook, YouTube и Instagram играют решающую роль в быстром и широкомасштабном распространении информации. Наиболее негативными последствиями дезинформации в области здравоохранения являются увеличение количества вводящих в заблуждение или неверных интерпретаций имеющихся данных, воздействие на психическое здоровье, нерациональное использование ресурсов в сфере здравоохранения и усиление сомнений в необходимости вакцинации. Увеличение количества недостоверной информации в области здоровья задерживает оказание медицинской помощи и увеличивает количество проявлений ненависти и разногласий. Социальные сети также могут быть полезным инструментом для борьбы с дезинформацией во время кризисов. Во всех включенных обзорах подчеркивается низкое качество опубликованных исследований во время кризисов в области здравоохранения.

**Вывод:**

Имеющиеся данные свидетельствуют о том, что инфодемия во время чрезвычайных ситуаций в области здравоохранения оказывает неблагоприятное воздействие на общество. Необходимы многосекторальные действия по противодействию инфодемии и дезинформации в области здравоохранения, включая разработку правовой политики, создание и продвижение кампаний по повышению осведомленности, улучшение контента, связанного со здоровьем, в средствах массовой информации и повышение цифровой и медицинской грамотности населения.

## Introduction

During crises, such as infectious disease outbreaks and disasters, the overproduction of data from multiple sources, the quality of the information and the speed at which new information is disseminated create social and health-related impacts.^1^^–^^3^ This phenomenon, called an infodemic, involves a torrent of online information containing either false and misleading information or accurate content.^4^

To tackle the production of misinformation (that is, false or inaccurate information deliberately intended to deceive) and disinformation (that is, deliberately misleading or biased information; manipulated narrative or facts; and propaganda) during recent pandemics or health emergencies, research on infodemics has increased. This research focuses on understanding the general effect of infodemics on society, dissemination patterns and delineating appropriate countermeasures policies.^5^^–^^7^ Several studies are analysing the effects of infodemics and misinformation and how societal behaviours are affected by that information.^8^^–^^10^ Particularly, evaluating infodemic-related concepts, such as impact on humans’ lives and communities, frequency and most common sources to widespread unreliable data, using comprehensive and evidence-based criteria, has gained more attention.^11^ Therefore, assessing how infodemics and health misinformation affect public health and identifying the availability and quality of evidence-based infodemic characteristics is timely and pertinent to inform appropriate management of its potential harms and support the development of monitoring guidelines.

We conducted a systematic review of reviews to collate, compare and summarize the evidence from the recent infodemics. To improve and guide the infodemic management, we designed our study to identify current opportunities, knowledge gaps and challenges in addressing the negative effects of the dissemination of health misinformation on public health.

## Methods

We registered our systematic review in PROSPERO (CRD42021276755). The review adheres to the Preferred Reporting Items for Systematic reviews and Meta-Analyses 2020 and the Quality of Reporting of Meta-analyses statement.^12^^,^^13^

We explored the following research questions: (i) To what extent are evidence-based studies addressing peculiarities and singularities associated with infodemics? (ii) What type of information on the topic of infodemics are published in systematic reviews? (iii) What main challenges, opportunities and recommendations addressing infodemics did systematic review authors highlight? and (iv) What is the methodological and reporting quality of published systematic reviews conveying research questions related to infodemic?

### Inclusion criteria

We used a published definition of systematic reviews^14^ and included a systematic review or mini-reviews if: (i) the search strategy was conducted at least in two databases; (ii) the study had at least two authors; and (iii) the study comprehensively presented a methods section or description of inclusion and exclusion criteria. We only included systematic reviews that directly analysed the available evidence correlated to infodemics, misinformation, disinformation, health communication, information overload and fake news (defined as: purposefully crafted, sensational, emotionally charged, misleading or totally fabricated information that mimics the form of mainstream news). We excluded preprints, unpublished data and narrative or literature reviews.

### Search methods

With an information specialist, we designed the search strategy using medical subject headings and specific keywords (Box 1). We had no restriction on publication date or languages. We searched five databases (MEDLINE®, Embase®, Cochrane Library of Systematic Reviews, Scopus and Epistemonikos), explored the reference lists of the included studies and searched for potential review protocols registered on PROSPERO. We first conducted the search on 4 November 2021 and we re-ran the search on 6 May 2022.

Box 1 **Search strategy for the systematic review on infodemics and health misinformation**

#1 Communication OR consumer health information OR information dissemination OR health literacy

#2 (infodemic* OR misinformation OR disinformation OR disinformation OR information dissemination OR information sharing* OR information overload) OR (fake new* OR influencer* OR conspirac* OR hate* OR infoxication) OR ((viral AND (news OR social media OR media)) OR (consumer health information OR health literacy OR health information literacy)

#3 - #1 OR #2

#4 Systematic review as topic OR PT Systematic review OR AB “systematic review” OR TI “systematic review”

#5 - #3 AND #5

After removing duplicates, two authors independently screened title, abstract and full-text of articles and included eligible articles for evaluation. An independent third author resolved any disagreements. We performed the screening process in Covidence (Covidence, Melbourne, Australia).

### Data collection and analysis

Two independent researchers extracted the general characteristics of each study and classified them into six major categories: (i) reviews evaluating negative effects of misinformation; (ii) reviews assessing the sources of health misinformation and the most used platforms; (iii) reviews evaluating the proportion of health-related misinformation on social media; (iv) reviews evaluating the beneficial features of social media use; (v) reviews associated with corrective measures against health misinformation; and (vi) reviews evaluating characteristics associated with studies’ quality. We clustered systematic reviews based on similar properties associated with the stated objective and the reported outcomes. Although infodemics were primarily defined as the overabundance of information, usually with a negative connotation, we decided to report data from systematic reviews which also described the potential beneficial effects of the massive circulation of information and knowledge during health emergencies. We summarized challenges and opportunities associated with infodemics and misinformation. A third author verified the retrieved data and another author resolved any inter-reviewer disagreement.

### Assessment of methodological quality

Two authors independently appraised the quality of included systematic reviews using the AMSTAR 2 tool, containing 16 domains.^15^ We rated each categorical domain using the online platform and obtained an overall score of critical and non-critical domains. Inter-rater discrepancies were resolved through discussion. We calculated inter-rater reliability with a Cohen’s *κ* and we classified reliability as adequate if *κ* > 0.85.

### Data synthesis

We synthesized the characteristics of included reviews, reporting their primary outcomes categorized by the similarity of the review question or results. Additionally, we created summary tables showing current evidence and knowledge gaps. We rated the certainty of the evidence through an adapted version of the Grading of Recommendations Assessment, Development and Evaluation approach for the defined primary outcomes.^16^^,^^17^

## Results

**Fig. 1:**
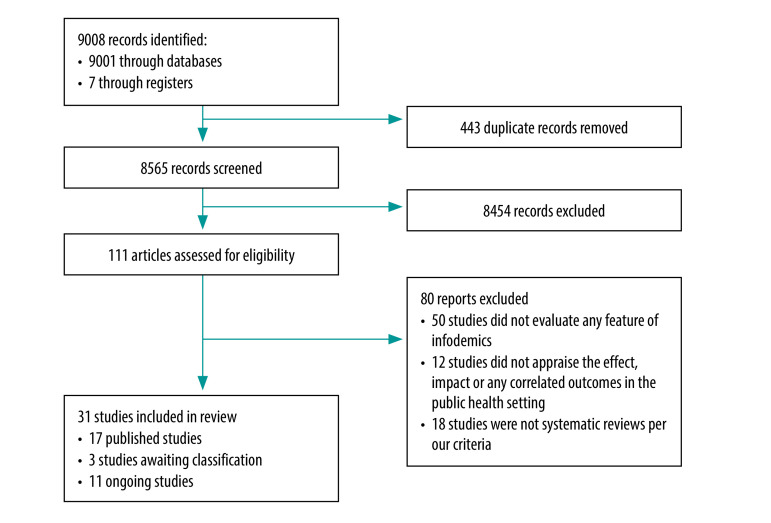
Selection of systematic reviews on infodemics and health misinformation

Note: We denoted studies for which we were unable to retrieve the full text, even after an exhaustive search, as awaiting classification. Therefore, these studies were considered in the included studies section based on the inclusion criteria. However, we cannot guarantee if these records are definitely eligible for inclusion.

We identified 9008 records and after removing 443 duplicates, we screened 8565 studies of which 111 were eligible for full-text assessment. Of these, we excluded 80 studies (available in the data repository).^18^ We included 31 systematic reviews, of which 17 studies were published between 2018 and 2022,^19^^–^^35^ three awaiting classification (we were unable to retrieve full text during our review)^36^^–^^38^ and 11 ongoing reviews (Fig. 1).^39^^–^^49^ Inter-rater reliability was high (*κ* = 0.9867).

Out of 17 published systematic reviews, 14 were published after the severe acute respiratory syndrome coronavirus 2 (SARS-CoV-2) outbreak.^19^^–^^35^ The published reviews included 1034 primary studies covering 12 infectious diseases and three major topics (vaccination hesitancy, disaster communication and disease outbreaks) related to infodemics, misinformation, disinformation, fake news or any other variation of these terms (Table 1). The included reviews covered 19 official scientific databases.

**Table 1:** Summary of included reviews on infodemics and health misinformation

Review, year	No. of databases (names)	No. of studies (study types)	Study objective
**Published systematic reviews**			
Abbott et al., 2022^19^	8 (PubMed®, Epistemonikos, Cochrane Library of Systematic Reviews, Cochrane COVID-19 Study Register, Embase®, CINAHL, Web of Science and WHO databases)	280 (systematic reviews, overviews and meta-analysis)	To map the nature, scope and quality of evidence syntheses on COVID-19 and to explore the relationship between review quality and the extent of researcher, policy and media interest
Alvarez-Galvez et al., 2021^21^	7 (Scopus, MEDLINE®, Embase®, CINAHL, Sociological Abstracts, Cochrane Library of Systematic Reviews and grey literature^a^)	42 (quantitative and qualitative studies and mixed-methods studies)	To identify the factors that make possible the spread of medical and health misinformation during outbreaks and to reveal the needs and future directions for the development of new protocols that might contribute to the assessment and control of information quality in future infodemics
Aruhomukama & Bulafu, 2021^29^	2 (PubMed® and CINAHL)	10 (quantitative and qualitative studies)	To interrogate and integrate knowledge levels and media sources of information findings of the studies on knowledge, attitudes, perceptions and practices towards COVID-19 done in low- and middle-income countries in Africa
Bhatt et al., 2021^30^	4 (MEDLINE®, Embase®, Cochrane Databases and Google)	5 (quantitative and qualitative studies)	To assess the current use of social media in clinical practice guidelines dissemination across different medical specialties
Eckert et al., 2018^23^	8 (PubMed®, Web of Science, CINAHL, CINAHL Complete, Communication and Mass Media Complete, PsychInfo®, WHO databases and Google Scholar) along with social media companies' reports	79 (quantitative and qualitative studies and case studies)	To conduct a systematic review on the extant literature on social media use during all phases of a disaster cycle
Gabarron et al., 2021^20^	5 (PubMed®, Scopus, Embase®, PsychInfo® and Google Scholar)	22 (mixed-methods studies)	To review misinformation related to COVID-19 on social media during the first phase of the pandemic and to discuss ways to counter misinformation
Gunasekeran et al., 2022^31^	3 (PubMed®, including MEDLINE® and Institute of Electrical and Electronics Engineers Xplore)	35 (quantitative and qualitative studies)	To highlight a brief history of social media in health care and report its potential negative and positive public health impacts
Lieneck et al., 2022^32^	2 (EBSCO host and PubMed®)	25 (quantitative and qualitative studies)	To identify common facilitators and barriers in the literature which influence the promotion of vaccination against COVID-19
Muhammed & Mathew, 2022^33^	7 (Web of Science, ACM digital library, AIS electronic library, EBSCO host, ScienceDirect, Scopus and Springer link)	28 (quantitative and qualitative studies)	To identify relevant literature on the spread of misinformation
Patel et al., 2020^26^	6 (all databases of Web of Science, PubMed®, ProQuest, Google News, Google and Google Scholar)	35	To canvas the ways disinformation about COVID-19 is being spread in Ukraine, so as to form a foundation for assessing how to mitigate the problem
Pian et al., 2021^34^	12 (PubMed®, CINAHL Complete, PsychInfo®, Psych Articles, ScienceDirect, Wiley Online Library, Web of Science, EBSCO, Communication & Mass Media Complete Library, Information Science & Technology Abstracts and Psychology & Behavioral Sciences Collection)	251 (quantitative and qualitative studies)	To synthesize the existing literature on the causes and impacts of the COVID-19 infodemic
Rocha et al., 2021^35^	3 (MEDLINE®, Virtual Health Library and Scielo)	14 (quantitative and qualitative studies)	To evaluate the impact of social media on the dissemination of infodemic knowing and its impacts on health
Suarez-Lledo & Alvarez-Galvez, 2021^24^	2 (MEDLINE® and PREMEDLINE)	69 (policy briefs and technical reports)	To identify the main health misinformation topics and their prevalence on different social media platforms, focusing on methodological quality and the diverse solutions that are being implemented to address this public health concern
Tang et al., 2018^25^	5 (PubMed®, PsychInfo®, CINAHL Plus, ProQuest® and Communication Source)	30 (quantitative and qualitative studies)	To better understand the status of existing research on emerging infectious diseases communication on social media
Truong et al., 2022^27^	4 (PsychInfo®, MEDLINE®, Global Health and Embase®)	28 (quantitative and qualitative studies)	To examine the factors that promote vaccine hesitancy or acceptance during pandemics, major epidemics and global outbreaks
Walter et al., 2021^22^	7 (Communication Source, Education Resources Information Center, Journal Storage, MEDLINE®, ProQuest, PubMed® and Web of Science)	24 (quantitative and qualitative studies)	To evaluate the relative impact of social media interventions designed to correct health-related misinformation
Wang et al., 2019^28^	5 (PubMed®, Cochrane Library of Systematic Reviews, Web of Science, Scopus and Google Scholar)	57 (mixed-methods studies)	To uncover the current evidence and better understand the 47 mechanisms of misinformation spread
**Ongoing systematic reviews, preprints or awaiting classification**			
Adu et al., 2021^39^	NA	NA	To estimate COVID-19 vaccine uptake and hesitancy rates for before-and-after the first COVID-19 vaccine was approved by FDA
Dong et al., 2022^40^	NA	NA	To review and synthesize the findings from qualitative studies conducted in different countries on the emergence, spread and consequences of false and misleading information about the pandemic
Fazeli et al., 2021^37^	NA	NA	Awaiting classification (limited access to the full-text file)
Gentile et al., 2021^36^	NA	NA	Awaiting classification (limited access to the full-text file)
Goldsmith et al., 2022^41^	NA	NA	To determine the extent and nature of social media use in migrant and ethnic minority communities for COVID-19 information and implications for preventative health measures including vaccination intent and uptake
Hilberts et al., 2021^42^	NA	NA	To establish the risk of health misinformation in social media to public health
Karimi-Shahanjarin et al., 2021^43^	NA	NA	To identify what initiatives and policies have been suggested and implemented to respond to and alleviate the harm caused by misinformation and disinformation concerning COVID-19
McGowan & Ekeigwe, 2021^44^	NA	NA	To assess if exposure to misinformation or disinformation influence health information-seeking behaviours
Pauletto et al., 2021^45^	NA	NA	To evaluate what are pros and cons of using social media during the COVID-19 pandemic
Pimenta et al., 2020^46^	NA	NA	To gather evidence on the impact of information about COVID-19 on the mental health of the population
Prabhu & Nayak, 2021^47^	NA	NA	To appraise what are the effects of the COVID-19 media based infodemic on mental health of general public
Trushna et al., 2021^48^	NA	NA	To undertake a mixed-methods systematic review exploring COVID-19 stigmatization, in terms of differences in experience and/or perception of different population sub-groups exposed to COVID-19, its mediators including media communications, coping strategies adopted to deal with such stigmata and the consequences in terms of health effects and health-seeking behaviour of affected individuals
Vass et al., 2022^38^	NA	NA	Awaiting classification (limited access to the full-text file)
Zhai et al., 2021^49^	NA	NA	To provide an overview of the current state of research concerning individual-level psychological and behavioural response to COVID-19-related information from different sources, as well as presenting the challenges and future research directions

COVID-19: coronavirus disease 2019; FDA: Food and Drug Administration; NA: not applicable; WHO: World Health Organization.

^a^ There was an inconsistency between the used databases provided in the study’s abstract and those presented in the methods section. We considered the databases shown in the methods section.

The main outcomes, categorized in six themes, are summarized in Box 2 and by study in Table 2. Below we describe the outcomes, by theme, in more detail.

Box 2 **Summary of studies’ outcomes**

**Effects of infodemics, misinformation, disinformation and fake news (10 studies)**

- Reduce patients’ willingness to vaccinate
- Obstruct measures to contain disease outbreaks
- Instigate the physical interruption of access to health care
- Amplify and promote discord to enhance political crisis
- Increase social fear, panic, stress and mental disorders
- Enhance misallocation of resources
- Weak and slow countermeasures interventions
- Exacerbate poor quality content creation

**Source of health misinformation propagation (six studies)**

- Social media platforms are associated as a potential source of promotion of anecdotal evidence, rumours, fake news and general misinformation
- Twitter, Facebook, Instagram and blogs play an important role in spreading rumours and speculating on health-related content during pandemics
- Digital influencers or well-positioned individuals acts as distractors or judges in social networks
- Closed communication within online communities can be used to propagate and reverberate unreliable health information
- Misinformation can be derived from poor quality scientific knowledge

**Proportion of health misinformation on social media (four studies)**

- Health misinformation in posts on social media is common (1–51% on posts associated with vaccine, 0.2–28.8% on posts associated with COVID-19 and 4–60% for pandemics)
- Approximately 20–30% of the YouTube videos about emerging infectious diseases contain inaccurate or misleading information

**Adequate use of social media (eight studies)**

- Social media platforms and traditional media might be useful during crisis communication and during emerging infectious disease pandemics, regardless of the geographical settings
- Using social media properly, infosurveillance can be highly functional in tracking disease outbreaks
- Social media can improve knowledge acquisition, awareness, compliance and positive behaviour towards adherence to clinical infection protocols and behaviours

**Corrective interventions (four studies)**

- Correcting misinformation delivered by health professionals is harder than information delivered by health agencies
- Misinformation corrected by experts is more effective than when corrected by non-experts
- The effectiveness of correcting misinformation using text or images is similar
- Use of refutational messages, directing the user to evidenced-based information platforms, creation of legislative councils to battle fake news and increase health literacy are shown to be effective countermeasures

**Overall quality of publications during infodemics (three studies)**

- Most studies published during an infodemic are of low methodological quality
- There is a substantial overlap of published studies addressing the same research questions during an infodemic

COVID-19: coronavirus disease 2019. Note: Grading of evidence is presented in Table 4.

**Table 2:** Summary of findings

Review (disease and/or condition)	Summary of findings
Abbott et al. (SARS-CoV-2)^19^	• Overlap of published studies related to SARS-CoV-2 between 10 and 15 June 2020 (for example, 16 reviews addressed cerebrovascular-related comorbidities and COVID-19, as well as 13 reviews evaluating the broad topic related to chloroquine and hydroxychloroquine). • Despite the rapid pace to gather evidence during the pandemic, published studies were lacking in providing crucial methodological and reporting components (for instance, less than half of included studies critically appraised primary studies, only a fifth of included reviews had an information specialist involved in the study, and only a third registered a protocol). • Lack of transparent searching strategies and a lack of assessment and consideration of potential limitations and biases within the included primary studies limits the validity of any review and the generalizability of its findings. • The lack of prior registration of a review protocol was directly associated with poor quality of evidence. • Even though some reviews had been considered of low methodological quality, social media and academic circles highlighted these studies.
Alvarez-Galvez et al. (SARS, H1N1 and H7N9 influenza viruses, Ebola virus, Zika virus, Dengue virus, generic diseases, poliomyelitis)^21^	• The authors identified five determinants of infodemics: (i) information sources; (ii) online communities' structure and consensus; (iii) communication channels; (iv) message content; and (v) health emergency context. • Health misinformation can propagate through influencers, opinion leaders, or well-positioned individuals that may act as distractors or judges in specific social networks and certain philosophies and ideologies have higher impact on low health-literate individuals. • Misinformation is frequently derived from poor quality scientific knowledge. • Traditional media can contribute to the wrong interpretation of existing scientific evidence. • Opinion polarization and echo chamber effects can increase misinformation due to the homophily between social media users. For instance, considering Facebook and Twitter, people tend to spread both reliable and untrusting information to their networks. • Misleading health contents propagate and reverberate among closed online communities which ultimately reject expert recommendations and research evidence. • Although social media platforms offer opportunities for specialists to convey accurate information, they also offer other non-specialists opportunities to counter this with the spread of misinformation and exacerbating outrage. • Mass media can propagate poor-quality information during public health emergencies: it seems to be an ideal channel to spread anecdotal evidence, rumours, fake news and general misinformation on treatments and existing knowledge about health topics. • Included studies demonstrated that the number of high-quality platforms with health-related content is limited and these have several issues (e.g. language restriction and failure to publicize). • Alarmist, misleading, shorter messages and anecdotal evidence seem to have a stronger impact on the spread of misinformation.
Aruhomukama & Bulafu (SARS-CoV-2)^29^	• Forty per cent of included studies showed that nearly all of the respondents had heard about COVID-19, while only one included study stated that participants had inadequate knowledge of COVID-19. • Participants reported that social media and local television and radio stations were their major source of information with regards to COVID-19. • In two studies, participants confirmed that their family members and places of worship (churches and mosques) were the main information resource. • Authors also suggest the SARS-CoV-2 pandemic has not dramatically affected Africa due to high levels of knowledge, positive attitudes and perceptions and good practices for infection control. • Authors also suggest the need for health agencies to trail misinformation related to COVID-19 in real time, and to involve individuals, communities and societies at large to demystify misinformation.
Bhatt et al. (neurological, gastrointestinal, cardiovascular and urological diseases)^30^	• Based on included studies, there was a significant improvement in knowledge, awareness, compliance, and positive behaviour towards clinical practice guidelines with the use of social media dissemination compared to standard methods. • Included studies found that social media has a crucial role in rapid and global information exchange among medical providers, organizations and stakeholders in the medical field, and its power can be harnessed in the dissemination of evidence-based clinical practice guidelines that guide physicians in practice. • Methods for data dissemination varied from systematic tweets on clinical practice guidelines at regular intervals using a social media model, audio podcasts and videos on YouTube. Studies also found that the mixture of written text and visual images on social media with links to medical guidelines, multimedia marketing, and production company-led paid social media advertising campaigns also has great effect in improving knowledge. • The review did not find any standardized method of analysing the impact of social media on clinical practice guidelines dissemination as the methods of dissemination were highly variable.
Eckert et al. (disaster communication)^23^	• Each social media platform used for information streaming is beneficial during crisis communication for government agencies, implementing partners, first responders, and the public to create two-way conversations to exchange information, create situational awareness and facilitate delivery of care. • Social media mostly focused on spreading verified information and eliminating rumours via crowd-sourced peer rumour control, sometimes combined with quick and effective myth-busting messages by government officials. • Social media must be combined with other channels, especially with messages on traditional news media as they still have high credibility and were most often referenced on Twitter and social media. • Social media should be used by agencies, first responders and the public to monitor public reactions during a crisis, to address the public, create situational awareness, for citizen's peer-to-peer communication and aid, and to solicit responses from the ground (specifically of those individuals who are directly affected by a disaster). • Social media can also be effective during the preparation phase as it can train potentially vulnerable populations who would need to be evacuated. • Social media should be used to send and receive early warning messages during all phases of the disaster, to share information on the situation on the ground during onset and containment phases, and to inform friends, families and communities about aid, food, and evacuees during the containment phase. Twitter was suggested as a tool to map in real time the spread of floods and assess damage during a disaster.
Gabarron et al. (SARS-CoV-2)^20^	• Six of 22 studies that reported the proportion of misinformation related to SARS-CoV-2 showed that misinformation was presented on 0.2% (413/212 846) to 28.8% (194/673) of posts. • Eleven studies did not categorize the specific type of COVID-19-related misinformation, nine described specific misinformation myths and two categorized the misinformation as sarcasm or humour related to the disease. • Four studies examined the effect of misinformation (all reported that it led to fear and panic). One of the four reported that misallocation of resources and stress experienced by medical workers were also possible consequences of misinformation. • One study reported that approximately 46.8% (525/1122) of survey respondents were tired of COVID-19 being the main theme across all media. • Four studies mentioned increasing the health literacy of social media users. • These studies highlighted the need to educate social media users on how to determine what information is reliable and to encourage them to assume personal responsibility for not circulating false information.
Gunasekeran et al. (SARS-CoV-2 and COVID-19)^31^	• The exponential potential of social media for information dissemination has been strategically used for positive impact in the past. They can be applied to reinvigorate public health promotion efforts and raise awareness about diseases. • The epidemiological value of social media applications includes surveillance of information, disease syndromes and events (outbreak tracing, needs or shortages during disasters). • To draw attention to accurate information, social media seems to present a potential tool for governments to (i) rapidly assess public reaction to an outbreak, (ii) identify critical time points and topics that need to be addressed, and (iii) rapidly disseminate vital public health communication during outbreaks. • The review suggested that infoveillance (i.e. information surveillance) is the detection of events using web-based data, which can be faster than traditional surveillance methods. Earlier studies have successfully illustrated the use of microblogs and users’ geographical locations to track infectious disease outbreaks in many countries. • Although social media has the potential for positive public health utility, it can also amplify poor quality content. Public fear and anxiety are known to be heightened by sensational reporting in the media during outbreaks, a phenomenon heightened by the ease of sharing on social media. • Despite the negative impact of social media in propagating infodemics, it also provides a reservoir of user-generated content as individuals share a range of topics from emotions to symptoms. • Social media has also been applied as a tool for grassroots health promotion initiatives.
Lieneck et al. (SARS-CoV-2 and COVID-19)^32^	• One of the largest barriers to vaccine promotion through social media during the COVID-19 pandemic has been misinformation spread on social media. • Many sites such as Twitter and Facebook do not directly monitor these falsehoods which can be detrimental to the acceptance of the COVID-19 vaccine and putting a stop to the virus. • As vaccine hesitancy grows, social media can either be a tool to encourage greater protection via the COVID-19 vaccine or continue to fill knowledge gaps with misinformation preventing vaccination. • During the COVID-19 pandemic specifically, studies show that social media is contributing to the spread of misinformation about the vaccine, and that individuals who were hesitant about the vaccine were more likely to only use social media as their source of news. • Due to a lack of regulation of social media, a lot of vaccine scepticism can spread via such channels. This lack can particularly affect the COVID-19 vaccine acceptance rate among individuals. • As social media continues to rise in popularity, it has the potential to be an effective source of public health information that is accessible and up to date. • Social media platforms are increasing their efforts to reduce the amount of misinformation by limiting the untrue information and directing people to evidence-based websites. One potential strategy for controlling the spread of misinformation suggests the use of elaborated refutational messages, which can reduce misperceptions because they help people understand the flaws of misinformation.
Muhammed & Mathew (COVID-19, Australian Bushfire and the USA elections)^33^	• When a crisis occurs, affected communities often experience a lack of localized information needed for them to make emergency decisions. • Information overload and information dearth are the two concerns that interrupt the communication between the affected community and a rescue team. • Dread rumour looks more trustworthy and more likely to get viral. Dread rumour was the cause of violence against a minority group during COVID-19. • Political misinformation has been predominantly used to influence the voters. Misinformation spreads quickly among people who have similar ideologies. • Confirmation bias has a dominant role in social media misinformation related to politics. Readers are more likely to read and engage with the information that confirms their pre-existing beliefs and political affiliations and reject information that challenges it. • Health misinformation could delay proper treatment, which could further deteriorate patients’ health status and affect relevant outcomes, including mortality rate. • In the context of emergency situations (unforeseen circumstances), the credibility of social media information has often been questioned mostly by users, lawmakers, health professionals and the media. • The broadcasting power of social media and re-sharing of misinformation could weaken and slow down rescue operations. • Discourse on social media misinformation mitigation has resulted in prioritization of strategies such as early communication from the officials and use of scientific evidence. • Rumour correction models for social media platforms employ algorithms, mathematical simulations and crowdsourcing. • Studies on controlling misinformation in the public health context showed that the government could also seek the help of public health professionals to mitigate misinformation
Patel et al. (SARS-CoV-2)^26^	• The disinformation related to crisis communication about COVID-19 was focused on eroding trust in the government’s response and the accuracy of the official health messaging or misleading the public about accessing and receiving resources or support. • Decreased trust in governments and public health systems leads to disregard for the official health advice and impacts the population’s medical decision-making, often with serious detrimental effects. • The combination of actions to decrease trust in governments and health-related organizations are compounded in disadvantaged or vulnerable populations, such as those living in poverty, regions of conflict or in areas with poor infrastructure. The communication crisis faced during the COVID-19 pandemic can be attributed to a legacy of government mistreatment and a general lack of access to reliable information, which strengthens the impact of disinformation campaigns. • The malicious intent and execution of disinformation campaigns in Ukraine were intended to amplify and promote discord to create a political impact in Ukraine, particularly in the context of the ongoing war. • Disinformation instigated the physical interruption of access to health care.
Pian et al. (COVID-19)^34^	• Social media use and low level of health and/or eHealth literacy were identified as the major causes of the infodemic. • There is a pattern of spiral-like interactions between rumour-spreading and psychological issues. Integrating psychological variables with models of rumour-sharing behaviour might be beneficial. • Multidisciplinary empirical studies should be conducted to validate the effectiveness of countermeasures applied to multiple groups (such as low level of health/eHealth literacy, social media/mass media platforms, governments, and organizations). Even if the countermeasures seem logical, how effective they are when applied in different contexts (e.g. different geographical regions, user profile, social media platform, etc.) need to be investigated. • One of the major causes of the infodemic is social media use, although social media can play a positive or negative role. • The rapid publication of editorials, commentaries, viewpoints and perspectives are also mentioned by the authors of the review to be the major cause of the infodemic, due to its low level of certainty and evidence. • Negative impacts were identified and related to the infodemic, including public psychological issues, breakdown of trust, inappropriate protective measures, panic purchase and the global economy. • The authors proposed various countermeasures against the COVID-19 infodemic, which were grouped into the following categories: countermeasure strategies for a low level of health and/ or eHealth literacy, social media/mass media platforms, governments, and organizations, risk communication and health information needs and seeking.
Rocha et al. (COVID-19)^35^	• Infodemic can cause psychological disorders and panic, fear, depression and fatigue. • Many occurrences were false news masquerading as reliable disease prevention and control strategies, which created an overload of misinformation. • Different age groups interact differently with the fake news propagated by social media. A specific focus should be given to people older than 65 years as they usually have limited skills managing social media systems. • Social media has contributed to the spread of false news and conspiracy theories during the COVID-19 pandemic. • Infodemic is part of people’s lives around the world, causing distrust in governments, researchers and health professionals, which can directly impact people’s lives and health. • During the COVID-19 pandemic, the disposition to spread incorrect information or rumours is directly related to the development of anxiety in populations of different ages.
Suarez-Lledo & Alvarez-Galvez (vaccines, smoking, drugs, noncommunicable diseases, COVID-19, diet and eating disorders)^24^	• Health topics were ubiquitous on all social media platforms included in the study. However, the health misinformation proportion for each topic varied depending on platform characteristics. • The proportion of health misinformation posts was dependent on the topic: vaccines (32%; 22/69), drugs or smoking issues (22%; 16/69),^a^ noncommunicable diseases (19%; 13/69), pandemics (10%; 7/69), eating disorders (9%; 6/69) and medical treatments (7%; 5/69). • Twitter was the most used source for work on vaccines (14%; 10/69), drugs or smoking products (14%; 10/69), pandemics (10%; 7/69) and eating disorders (4%; 3/69). For studies on noncommunicable diseases (13%; 9/69) or treatments (7%; 5/69), YouTube was the most used social media platform. • Health misinformation was most common in studies related to smoking products, such as hookah and water pipes, e-cigarettes and drugs, such as opioids and marijuana. • Health misinformation about vaccines was also very common. Therefore, the potential effect on population health was ambivalent, that is, both positive and negative effects were found depending on the topic and on the group of health information seekers. • Authors identified social media platforms as a potential source of illegal promotion of the sale of controlled substances directly to consumers. • Misleading videos promoted cures for diabetes, negated scientific arguments or provided treatments with no scientific basis. • Although social media was described as a forum for sharing health-related knowledge, these tools are also recognized by researchers and health professionals as a source of misinformation that needs to be controlled by health experts.
Tang et al. (H1N1 and H7N9 influenza viruses, Ebola virus, West Nile virus, measles, MERS-CoV and enterohaemorrhagic *Escherichia coli*)^25^	• In general, approximately 65% (225/344) of videos contained useful information (either accurate medical information or outbreak updates) across different emerging infectious diseases, while the rest of videos contained inaccurate or misleading information. Whether misleading videos had a significantly higher number of views per day is unclear. • Independent users were more likely to post misleading videos and news agencies were more likely to post useful videos.
Truong et al. (vaccination, H1N1 and Ebola)^27^	• Lack of information and misinformation about vaccination against H1N1 influenced participants’ decision to vaccinate. • Lacking adequate information surrounding vaccination against H1N1 or encountering contradictory information from different sources can reduce an individual’s willingness to vaccinate. The lack of accurate information associated with vaccines would affect the population’s willingness to vaccinate against other infectious diseases (such as Ebola). • Although the internet can be a useful resource to spread vital public health information during a pandemic, a lack of clarity and consistency of information may deter people from vaccination. • People that do not have a comprehensive understanding of how vaccines work are unable to make informed and confident decisions about vaccination. Therefore, communicating information regarding vaccination in a clear and accessible manner to better educate people and overcome barriers to vaccination is essential.
Walter et al. (countermeasures against misinformation)^22^	• The meta-analysis showed that source of misinformation emerged as a significant moderator (*P*-value: 0.001). Specifically, correcting misinformation is more challenging when it is delivered by our peers (*d* = 0.24; 95% CI: 0.11–0.36) as opposed to news agencies (*d* = 0.48; 95% CI: 0.15–0.81). • The source of the correction played a significant role (*P*-value: 0.031), resulting in stronger effects when corrective messages were delivered by experts (*d* = 0.42; 95% CI: 0.28–0.55) compared with non-experts (*d* = 0.24; 95% CI: 0.13–0.34). • There was no significant difference (*P*-value: 0.787) between interventions that employed Facebook rather than Twitter. • Finally, the results suggest that it is more difficult to correct misinformation in the context of infectious disease (*d* = 0.28; 95% CI: 0.17–0.39) as opposed to other health-related issues (*d* = 0.55; 95% CI: 0.31–0.79). • The effects of myths about genetically modified produce, nutrition and reproductive health were more effectively attenuated by corrective interventions than misinformation about Zika virus, measles, HIV and other communicable diseases.
Wang et al. (vaccination, Ebola virus and Zika virus, along with other conditions and topics, including nutrition, cancer and smoking)^28^	• Misinformation is abundant on the internet and is often more popular than accurate information. • Most commonly health-related topics associated with misinformation are communicable diseases (30 studies), including vaccination in general (eight studies) and specifically against human papillomavirus (three studies), measles, mumps and rubella (two studies) and influenza (one study), as well as infections with Zika virus (nine studies), Ebola virus (four studies), MERS-CoV (one study) and West Nile virus (one study). • Misconceptions about measles, mumps and rubella vaccine and autism, in particular, remain prevalent on social media. • Other topics share scientific uncertainty, with the authorities unable to provide confident explanations or advice, as with newly emerging virus infections such as Ebola and Zika viruses.

CI: confidence interval; COVID-19: coronavirus disease 2019; H1N1: influenza A virus subtype H1N1; H7N9: Asian lineage avian influenza A H7N9; HIV: human immunodeficiency virus; MERS-CoV: Middle East respiratory syndrome coronavirus; SARS: severe acute respiratory syndrome; SARS-CoV-2: severe acute respiratory syndrome coronavirus 2.

^a^ Numbers are reported as given in original publication despite that the percentage is inconsistent with numerator and denominator.

### Negative effects of misinformation

Ten systematic reviews presented evidence of the negative effects associated with the dissemination of misinformation during an infodemic.^20^^,^^21^^,^^24^^,^^26^^,^^27^^,^^31^^–^^35^ Several of the consequences were linked to altering people’s attitude towards the situation: (i) distorting the interpretation of scientific evidence; (ii) opinion polarization and echo chamber effects (that is, the formation of groups of like-minded users framing and reinforcing a shared narrative); (iii) offering non-specialists’ opinions to counter accurate information; (iv) promoting fear and panic; (v) increasing mental and physical fatigue of population; and (vi) decreasing credibility of circulating information on different platforms during unforeseen circumstances. Infodemics could also decrease trust in governments and public health systems as well as in the government’s response and accuracy of the official health messaging. Other societal consequences could be amplifying and promoting discord to create a hostile political environment, increasing violence against ethnic and minority groups and affecting the global economy. Within the health system, infodemics could lead to (i) misallocation of resources and increasing stress among medical providers; (ii) decreased access to health care; (iii) increased vaccine hesitancy and conspiracy beliefs; (iv) increased illegal promotion of the sale of controlled substances; and (v) delayed delivery of high-quality care and proper treatment to patients, which could further have a negative effect on public health-care systems.

### Sources of health misinformation

Six reviews reported potential links between misinformation and sources of misinformation.^21^^,^^24^^,^^28^^,^^32^^,^^34^^,^^35^ All reviews emphasized that mass media can propagate poor-quality health information during public health emergencies, particularly through social media. Authors of the systematic reviews highlighted that health misinformation can be quickly propagated through media posts and videos, usually circulated among closed online groups, significantly influencing individuals with low health literacy and elderly patients.^21^^,^^34^^,^^35^ Similarly, two reviews found that social media networks were often identified as a source of illegal or inappropriate promotion of health misinformation, including the sale of controlled substances.^24^^,^^32^ One review tracked the main sources of health-related misinformation spreading on social media during infectious disease outbreaks worldwide, noting that the primary sources of misinformation are groups against immunization, online communication groups (such as WhatsApp groups and Facebook communities) and pharmaceutical and marketing industries, who could favour conspiracy theories.^28^

### Proportion of health-related misinformation

Four reviews evaluated the proportion of health misinformation on different social media platforms.^20^^,^^24^^,^^25^^,^^28^ In a meta-analysis, the proportion ranged from 0.2% (413/212 846) to 28.8% (194/673) of posts.^20^ Similarly, a review identified that the proportion of the literature containing health misinformation is dependent on the topic, which were articulated in six categories (vaccines had the highest proportion, 32%; 22/69, whereas medical treatments had the lowest, 7%; 5/69).^24^ One review identified 47 mechanisms driving misinformation spread.^28^ The authors also argued that misconceptions about vaccine administration in general and about infectious diseases (45 studies out of 57) and chronic noncommunicable diseases (8 studies out of 57) are highly prevalent on social media; however, the review lacks comprehensive presentation of epidemiologically-relevant data.^28^ Additionally, authors of a review estimated around 20% to 30% of YouTube videos about emerging infectious diseases contained inaccurate or misleading information.^25^

### Beneficial features of social media use

Although infodemics are often associated with negative impacts, eight reviews reported positive outcomes related to infodemics on social media during a pandemic.^21^^,^^23^^,^^24^^,^^29^^–^^33^ Social media can be used for crisis communication and management during emerging infectious disease pandemics, regardless of geographical location of the recipient of information. Furthermore, reviews found that dissemination of information on several social media platforms had significantly improved knowledge awareness and compliance to health recommendations among users.^21^^,^^30^^,^^31^ Notably, some authors also stressed the fact that social media created a positive health-related behaviour among general users compared with classic dissemination models.^21^^,^^30^^,^^31^ In particular, these platforms can be used for education, suggesting that social media could outrank traditional communication channels.^21^^,^^31^ Also, content created by professionals and published on social networks, especially YouTube, might serve the community with online health-related content for self-care or health-care training.^24^ Three reviews evaluated the effectiveness of social media platforms as vehicles for information dissemination during health-related disasters, including pandemics, as well as a tool to promote vaccination awareness. The reviews evidenced the effectiveness of social media platforms as an information surveillance approach, as these platforms could provide complementary knowledge by assessing online trends of disease occurrences, collecting and processing data obtained from digital communication platforms.^23^^,^^31^^,^^32^ Twitter and Facebook emerged as beneficial tools for crisis communication for government agencies, implementing partners, first responders and for the public to exchange information, create situational awareness, decrease rumours and facilitate care provision.^23^ Furthermore, these authors also argued that social media is a viable platform to spread verified information and eliminate unfiltered and unreliable information through crowd-sourced, peer-based rumour control (that is, technologies that network users can collaboratively implement for more effective rumour control).^23^^,^^31^^,^^32^

Interestingly, one study suggested that the use of social media to mitigate misinformation associated with health-related data might result not only from the prioritization of strategies taken by governmental and health authorities, but also from the economy sector, which also includes the information technology market, the media and knowledge-based services. Also, citizens’ intention to spread misinformation by using real information would ultimately serve as a natural controlling system.^33^

One systematic review evaluated the knowledge levels and media sources of information about coronavirus disease 2019 (COVID-19) in African countries^29^ and found that 40% (4/10) of studies reported that the participants used social media as their source to acquire information about COVID-19. Likewise, traditional communication channels (such as television and radio stations), family members and places of worship were also used to receive information about the disease. 

### Corrective measures against health misinformation

Four reviews evaluated the impact and effectiveness of social media interventions created to correct health-related misinformation.^22^^,^^32^^–^^34^ In general, eliminating health-related misinformation delivered by family or colleagues is more challenging than eliminating misinformation from organizations. Furthermore, evidence shows that a greater corrective effect occurs when content experts correct misconceptions compared with non-experts.^22^ In addition, authors of three reviews suggested redirecting users to evidence-based or well-founded websites, besides computer-based algorithms for rumour correction, as countermeasures to limiting the circulation of unreliable information.^32^^–^^34^ Early communication from health authorities and international health organizations plays an important role in providing misinformation mitigation.^22^^,^^32^^–^^34^

### Characteristics associated with studies’ quality

Three reviews reported results on methodological quality of included studies.^19^^,^^21^^,^^34^ Generally, studies related to SARS-CoV-2 and infodemics showed critical quality flaws. For example, 49% (138/280) of eligible studies critically appraised the quality of original records.^19^ In comparison, only 33.0% (29/88) of the studies reported the registration of a scientific protocol before the beginning of the study.^19^ Several systematic reviews did not consider in their final analysis and conclusion statements the limitations of each included study’s design.^20^^,^^22^^–^^25^^,^^28^^,^^29^^,^^31^ One study concluded that the spread of misinformation has been frequently derived from poor-quality investigations.^21^ Lastly, a large number of editorials, commentaries, viewpoints and perspectives were published since the onset of the COVID-19 pandemic; these types of articles are fast-tracked publications not based on new experimental and analytical data.^34^

### Methodological quality

When appraised using the AMSTAR 2 critical domains, 16 reviews (94.1%) scored as having critically low quality across most major domains.^19^^,^^21^^–^^35^ Only one review showed a moderate risk of bias for most domains (Table 3).^20^ Meta-analysis was conducted in only two reviews, which used appropriate statistical methods and considered the potential impact of risk of bias in each of the primary studies.^19^^,^^22^ The overall quality of the evidence is shown in Table 4. All themes had low quality, except the proportion of health-related misinformation which had very low quality of evidence.

**Table 3:** Quality of included systematic reviews on infodemics and health misinformation

Review	Methodological requirements met, by domain^a^																Overall quality
	1	2	3	4	5	6	7	8	9	10	11	12	13	14	15	16	
Abbott et al.^19^	Yes	Partly met	Yes	Partly met	Yes	No	No	Partly met	No	No	NA	NA	No	Yes	NA	Yes	Critically low
Alvarez-Galvez et al.^21^	Yes	No	Yes	No	Yes	No	No	Partly met	Yes	No	NA	NA	No	No	NA	Yes	Critically low
Aruhomukama & Bulafu^29^	Yes	No	No	No	Yes	No	No	Partly met	Yes	No	NA	NA	No	No	NA	Yes	Critically low
Bhatt et al.^30^	Yes	Partly met	Yes	Partly met	Yes	Yes	No	Partly met	Partly met	No	NA	NA	No	No	NA	Yes	Critically low
Eckert et al.^23^	Yes	No	Yes	Yes	No	No	No	Partly met	Yes	No	NA	NA	Yes	No	NA	No	Critically low
Gabarron et al.^20^	Yes	Partly met	Yes	Partly met	Yes	Yes	Yes	Yes	Yes	No	NA	NA	Yes	No	NA	Yes	Low
Gunasekeran et al.^31^	Yes	No	No	No	No	No	No	No	No	No	NA	NA	No	No	NA	Yes	Critically low
Lieneck et al.^32^	Yes	No	Yes	No	Yes	No	No	Partly met	Yes	No	NA	NA	No	No	NA	Yes	Critically low
Muhammed & Mathew^33^	Yes	No	Yes	Partly met	Yes	Yes	No	Partly met	Partly met	No	NA	NA	No	Yes	NA	Yes	Critically low
Patel et al.^26^	Yes	Partly met	Yes	Partly met	No	No	No	Partly met	No	No	NA	NA	No	No	NA	No	Critically low
Pian et al.^34^	Yes	No	Yes	Partly met	Yes	Yes	No	Partly met	No	Yes	NA	NA	Yes	No	NA	Yes	Critically low
Rocha et al.^35^	Yes	No	Yes	Partly met	No	No	No	Partly met	No	No	NA	NA	No	No	NA	No	Critically low
Suarez-Lledo & Alvarez-Galvez^24^	Yes	Partly met	Yes	Partly met	Yes	Yes	No	Partly met	Yes	No	NA	NA	No	No	NA	Yes	Critically low
Tang et al.^25^	Yes	Partly met	Yes	No	No	No	No	Partly met	No	No	NA	NA	No	No	NA	No	Critically low
Truong et al.^27^	Yes	No	No	No	Yes	Yes	No	No	No	No	NA	NA	No	No	NA	Yes	Critically low
Walter et al.^22^	Yes	Partly met	Yes	Partly met	Yes	Yes	No	Partly met	Yes	No	Yes	Yes	No	Yes	Yes	No	Critically low
Wang et al.^28^	Yes	Partly met	Yes	No	No	No	No	Partly met	No	No	NA	NA	No	No	NA	No	Critically low

NA: not applicable.

Note: We judged studies using the AMSTAR 2 tool.^15^ For domains rated NA, the review lacked a meta-analysis.

^a^ Domain 1: did the research questions and inclusion criteria for the review include the components of PICO (population, intervention, comparator and outcomes)? Domain 2: did the report of the review contain an explicit statement that the review methods were established before the conduct of the review and did the report justify any significant deviations from the protocol? Domain 3: did the review authors explain their selection of the study designs for inclusion in the review? Domain 4: did the review authors use a comprehensive literature search strategy? Domain 5: did the review authors perform study selection in duplicate? Domain 6: did the review authors perform data extraction in duplicate? Domain 7: did the review authors provide a list of excluded studies and justify the exclusions? Domain 8: did the review authors describe the included studies in adequate detail? Domain 9: did the review authors use a satisfactory technique for assessing the risk of bias in individual studies that were included in the review? Domain 10: did the review authors report on the sources of funding for the studies included in the review? Domain 11: if meta-analysis was performed did the review authors use appropriate methods for statistical combination of results? Domain 12: if meta-analysis was performed, did the review authors assess the potential impact of risk of bias in individual studies on the results of the meta-analysis or other evidence synthesis? Domain 13: did the review authors account for risk of bias in individual studies when interpreting/discussing the results of the review? Domain 14: did the review authors provide a satisfactory explanation for, and discussion of, any heterogeneity observed in the results of the review? Domain 15: if they performed quantitative synthesis did the review authors carry out an adequate investigation of publication bias (small study bias) and discuss its likely impact on the results of the review? Domain 16: did the review authors report any potential sources of conflict of interest, including any funding they received for conducting the review?

**Table 4:** Certainty of the evidence of main outcomes

Theme (no. of systematic reviews)	Certainty of the evidence (GRADE)					
	Methodological limitations^a^	Inconsistency^b^	Indirectness^c^	Imprecision^c^	Publication bias^d^	Overall quality
Negative effects of misinformation (10) ^20^^,^^21^^,^^24^^,^^26^^,^^27^^,^^31^^–^^35^	Critical	Not serious	NA	NA	Not serious	Low^e^
Source of health misinformation (6)^21^^,^^24^^,^^28^^,^^32^^,^^34^^,^^35^	Critical	Not serious	NA	NA	Not serious	Low^f^
Proportion of health-related misinformation (4)^20^^,^^24^^,^^25^^,^^28^	Critical	Serious	NA	NA	Not serious	Very low^g^
Beneficial features of social media use (8)^21^^,^^23^^,^^24^^,^^29^^–^^33^^h^	Critical	Not serious	NA	NA	Not serious	Low^f^
Corrective interventions against health misinformation (4)^22^^,^^32^^–^^34^	Critical	Not serious	Not serious	NA	Not serious	Low^f^
Characteristics associated with studies’ quality (3)^19^^,^^21^^,^^34^	Critical	Not serious	NA	NA	Not serious	Low^f^

GRADE: Grading of Recommendations Assessment, Development and Evaluation; NA: not applicable.

^a^ Methodological limitations were essentially associated with the overall AMSTAR 2 rating.

^b^ Inconsistency was judged by evaluating the consistency of the direction and primarily the difference in the magnitude of effects across studies (since statistical measures of heterogeneity are not available). As we did not find differing results for each outcome across included studies, we considered “not serious” risk for inconsistency, except for the “Proportion of health misinformation on social media,” as previously mentioned.

^c^ We did not downgrade the indirectness and imprecision domains for most outcomes because they were not referring to any applicable intervention on human beings or health condition. Thus, we marked it as “Not applicable.” For the only outcome associated with an intervention (Corrective interventions for health misinformation), we considered it to be at “not serious” risk of indirectness because there was an adequate association between the evidence presented and review question.

^d^ We downgraded the publication bias domain if the body of literature appears to selectively evidence a certain topic or trend from a specific outcome.

^e^ Downgraded due to methodological limitations of the included systematic reviews (most included reviews had an overall critically low methodological quality).

^f^ Downgraded due to methodological limitations of the studies (most included reviews had an overall critically low methodological quality).

^g^ Downgraded due to methodological limitations of the studies (most included reviews had an overall critically low methodological quality), and inconsistency (studies had widely differing estimates of the proportion, indicating inconsistency in reporting).

^h^ Social media also serves as a place where health-care professionals fight against false beliefs and misinformation on emerging infectious diseases.

Note: Low certainty by the GRADE Working Group grades of evidence: the summary rating of the included studies provides some indication of the likely effect. The likelihood that the effect will be substantially different is high. Very low certainty: the summary rating of the included studies does not provide a reliable indication of the likely effect. The likelihood that the effect will be substantially different is very high.

### Opportunities and challenges

We evaluated reported data on current opportunities and challenges associated with infodemics and misinformation that may impact society worldwide. A summary of the main opportunities for future research and challenges is presented in Box 3.

Box 3 **Summary of reported research opportunities and challenges**

**Future research**

- Future investigations should be performed to provide different aspects of the impact and reliability of SARS-CoV-2-related or any other health emergency information.
- There is a need to balance the gold standard systematic reviews with faster pragmatic studies.
- Studies need to evaluate effective methods to precisely combat the determinants of health misinformation during pandemics and subsequent infodemics across different social media platforms.
- Novel investigations could focus on creating a basis to conduct future studies (especially randomized trials) comparing the use of social media interventions with traditional methods in the dissemination of clinical practice guidelines.
- Future studies should assess the potential of social media use on the recovery and preparation phases of emergency events.
- Researchers could analyse communication patterns between citizens and frontline workers in the public health context, which may be useful to design counter-misinformation campaigns and awareness interventions.
- A multidisciplinary specialist team could concentrate on the analysis of governmental and organizational interventions to control misinformation at the level of policies, regulatory mechanisms and communication strategies.
- Studies should address the impact of fake news on social media and its influence on mental health and overall health.
- Future studies should examine how social media users process the emerging infectious diseases-related information they receive.
- Focus should be given to how users evaluate the validity and accuracy of such information and how they decide whether they will share the information with their social media contacts.
- Further interdisciplinary research should be warranted to identify effective and tailored interventions to counter the spread of health-related misinformation online.

**Challenges**

- Overlap of studies covering the same topic.
- Overall low quality of studies and the excessive and inordinate media attention given to these studies.
- Creation and use of reliable health-related information and scientific evidence considering real-time updates.
- Inadequate orientation of the population and medical providers into wrong pharmacological and non-pharmacological interventions.
- New trends in personal content creation are constantly emerging, such as TikTok, which represent new challenges for regulation.
- Further understanding the economic impact of misinformation, the difference in distribution of health misinformation in low- and high-income countries and the real impact of antivaccine activism groups.
- Decisive and pro-active actions are required from government authorities and social media developers to avoid the destruction of positive achievements that social media has already promoted.
- The difficulty of characterizing and evaluating the quality of the information on social media.

SARS-CoV-2: severe acute respiratory syndrome coronavirus 2.

## Discussion

In our study, most systematic reviews evaluating the social-, economic- and health-related repercussions of misinformation on social media noted a negative effect, either an increase in erroneous interpretation of scientific knowledge, opinion polarization, escalating fear and panic or decreased access to health care. Furthermore, studies reported that social media has been increasingly propagating poor-quality, health-related information during pandemics, humanitarian crises and health emergencies. Such spreading of unreliable evidence on health topics amplifies vaccine hesitancy and promotes unproven treatments. Moreover, reviews evidenced the low quality of studies during infodemics, mostly related to overlap between studies and minimal methodological criteria.

The increased spread of health-related misinformation in social and traditional media and within specific communities during a health emergency is accelerated by easy access to online content, especially on smartphones.^49^ This increased access and the rapid spreading of health-related misinformation through social media networks could have a negative effect on mental health.^50^^–^^53^

Although the number of studies evaluating variables associated with infodemics has risen, some variables still require further scientific exploration. For instance, some studies described the need for better methods of detecting health-related misinformation and disinformation, as the propagation methods are constantly evolving.^54^^–^^56^ Different initiatives have been used by individuals to reveal untrustworthy content, including website review, lateral reading approaches (that is, verifying content while you read) and emotion check analysis.^54^^–^^57^ However, no consensus exists on which method is more effective in battling unreliable content. Moreover, the techniques to build social media content that convey misinformation vary across different social media platforms and over time, even for the same platform.^58^^–^^60^ This variation implies the need to employ various multilingual detection and eradication techniques, which should be frequently updated to keep up with misinformation patterns. Evidence-based studies could evaluate the effectiveness of different misinformation detection models by comparing performance metrics and prediction values.^61^^–^^65^ Further priorities include recognizing methods to decrease the high-speed dissemination of misinformation and understanding the role social media plays in individuals’ real life after obtaining a certain content, information or knowledge from these platforms.

Only one review recommended legal measures against the publication of false or manipulated health information.^20^ Indeed, the discussion of this topic in the literature is controversial and polemical and is limited by the diversity of national legislative processes.^66^ For several jurists, the criminalization of intentionally sharing health misinformation acknowledges the wrongful violation of the right to life and liberty.^67^^–^^69^ Furthermore, proper attention must be paid to predatory journals that publish articles without minimum quality checks.^70^^,^^71^ For anti-criminalization supporters, creating policies controlling health misinformation and disinformation goes against freedom of speech and a free flow of information.^72^^,^^73^ Countermeasures not involving legal actions against health-related misinformation can be awareness campaigns for patients and health-care professionals, the creation and dissemination of easy-to-navigate platforms with evidence-based data, the improvement of health-related content in mass media by using high-quality scientific evidence, the increase of high-quality online health information and improved media literacy. Promoting and disseminating trustworthy health information is crucial for governments, health authorities, researchers and clinicians to outweigh false or misleading health information disseminated in social media. Another option is to use social media channels to counter the false or misleading information, which may require further studies to evaluate the best format for this outreach and which channels work best for different populations in different geographical and cultural settings.

This review has some limitations. First, we did not search for grey literature because studies have suggested that the searching efficiency and replicability of searches depends on geographical location and users’ content profile.^74^ Second, we assessed only systematic reviews and may have overlooked helpful non-systematic reviews. Nevertheless, by incorporating reviews published in scientific journals indexed in relevant databases, we obtained a comprehensive snapshot of the literature and we summarized reported gaps and implications for future research. Third, overviews of systematic reviews by nature depend on other researchers regarding inclusion criteria and methods of synthesizing data or outcomes. Thus, our conclusions may have been affected by by the bias that any systematic review author is potentially affected by. We took steps to minimize this bias, through creating a research protocol, assessing records by two authors and evaluating the quality of the evidence. Fourth, the quality of most included reviews were rated critically low due to non-adherence to important methodological features, a known issue of systematic reviews.^14^^,^^75^ Therefore, we advocate that researchers comply with reporting and executing guidelines for systematic reviews, which increases the completeness of reporting and assists with transparency and reproducibility of the study. Likewise, journals’ editors and reviewers should put into practice endorsed reporting guidelines which, although commonly displayed at journals’ interfaces, are not systematically employed during the evaluation process. However, we considered the low quality of included reports when interpreting and discussing the results.

Based on the available evidence, people are feeling mental, social, political and/or economic distress due to misleading and false health-related content on social media during pandemics, health emergencies and humanitarian crises. Although the literature exponentially increases during health emergencies, the quality of publications remains critically low. Future studies need improved study design and reporting. Local, national and international efforts should seek effective counteractive measures against the production of misinformative materials on social media. Future research should investigate the effectiveness and safety of computer-driven corrective and interventional measures against health misinformation, disinformation and fake news and tailor ways to share health-related content on social media platforms without distorted messaging.
